# Study on the effect of different contact times on the migration of heavy metals into different foodstuffs served in plastic cups

**DOI:** 10.1016/j.heliyon.2024.e31627

**Published:** 2024-05-21

**Authors:** B.M. Khaled, Adda Ann Sina, Md. Suman Rana, S. M. Shamiul Alam, Abdullah Al Numan, Maria Tabassum Shammi, Fatima Parvin, Tamanna Naznin, Md. Mozaffor Hossain, Refat Pervin Annana

**Affiliations:** a Department of Agro Product Processing Technology, Jashore University of Science and Technology, Jashore-7408, Bangladesh; b Bangladesh Food Safety Authority, Dhaka-1000, Bangladesh

**Keywords:** Heavy metals, Plastic cups, Migration, Food safety, Health risk, Risk assessment

## Abstract

Heavy metal concentrations of Pb, Cd, Cr, and Cu leaching from single-use plastic cups were identified, and the risks associated with them were assessed in real time (up to 10 min). All samples (tea, carbonated beverage, and lassi) were placed in disposable plastic cups and held for less than 1 min, 5 min, and 10 min, respectively. Prior to digestion, the solids were ashed in a muffle furnace at 550 °C for 30 min. The filtrates were then tested for heavy metals. The samples were all confirmed to be contaminated with heavy metals. Heavy metals leached from the plastic cups in the following order: Cu > Pb > Cr > Cd. The samples' HI values were less than one, hence there was no evidence of a non-carcinogenic risk. The ILCR values for this heavy metal contamination in samples exceed 10^−3^, indicating a high carcinogenic risk. Lassi poses the highest possible carcinogenic risk. A rise in temperature and a drop in pH also resulted in heavy metal migration. Heavy metal leaching from plastic cups poses a serious health risk due to its toxicity. The proposed legislation should prohibit or restrict the serving of warm and hot beverages in plastic cups.

## Highlights

• Plastic cups were detected with heavy metal.
• Heavy metals migrated from plastic cup into food products at different contact time.
• Temperature and pH variation of food and plastic influenced heavy metals leaching.
• Heavy metals leached from plastic cups in the following order: Cu > Pb > Cr > Cd.
• HI < 1 and ILCR>10^−3^ indicated no non-carcinogenic and significant carcinogenic risk.

## 1 Introduction

For decades, people have used plastics to package agricultural commodities and food items. Plastics are primarily consumed by packaging industries [1]. It replaced glass containers and cans because of their flexibility and availability. Plastics used in packaging are typically single-use plastics. Restaurants, roadside food vendors, tea shops, and other establishments use them as packaging material and utensils. Plastic boxes, pouches, spoons, plates, cups, and other items made of single-use plastics are common. Plastic cups have been popular for many years due to its light weight, transparency, and ease of transportation. During the COVID-19 pandemic, single-use plastic cups became popular for hygiene purposes. Currently, numerous food vendors provide tea, coffee, fizzy beverages, and milk products in plastic cups.

Various types of plastics undergo specialized additions such as additives and catalysts during their production. Because raw plastics are brittle, hard chemicals are usually added throughout the production process to impart specific attributes to the final product (such as elasticity, stability, color density, and transparency). Certain additives may prevent or delay the degradation of plastics [2]. Plastic additives and/or catalysts are potentially hazardous metal compounds that circulate in the environment as a result of the extensive use of plastic products. However, functional additives are the most common source of metals in plastics in general [3]. Typically, the polymer melt introduces additives as liquids or solids, with the latter forming small particles that scatter in matrix with typical sizes of a few micrometers or a few hundred nanometers [4]. These chemicals, known as heavy metals, have the potential to migrate into food if the packaging is not separated by a functional barrier during ambient packaging conditions [5].

Heavy metals present in plastic packaging have the capacity to leach into food, leading to the development of cancer and other significant health problems. Heavy metals are highly hazardous contaminants that are frequently found in the environment and pose a substantial risk to both people and other organisms [6]. Their functional technique is the inactivation of enzyme systems or crucial protein structures, ultimately leading to cellular dysfunction and death [7]. Exposure to lead can impair mental ability in youngsters, but it does not easily cross the blood-brain barrier in adults. Prolonged exposure to lead can result in memory impairment, decreased response speed, cognitive deterioration, and potentially even the development of cancer. Cadmium induces hypertension, neurological problems, osteoporosis, cardiovascular impairment, and carcinogenicity [8]. Prolonged exposure to antimony, chromium, and nickel might potentially lead to the development of cancer and developmental problems in both males and females [9]. Plastic, being one of the major sources, is indirectly condemned as it leaches heavy metals that are all carcinogenic to some extent. Food products like hot tea, carbonated beverages, and lassi are very popular among the local people of Bangladesh. They are consumed in tea stalls, restaurants, and coffee houses. Typically, they are presented in plastic cups. The rationale for selecting these three samples is mostly based on their consumption rate and availability in the context of Bangladesh.

Scientists around the globe are conducting research to figure out the causes and attributes of the leaching of heavy metals from plastics and plastic items. [10] reported the leaching rate of heavy metals like Cd, Pb, Sb in plastic containers from China is sufficiently high to pose carcinogenic risk. [11] stated that high temperature had a significant effect on time-dependent release of antimony in PET (Polyethylene Terephthalate) bottles. Recent studies reported by [12] investigated the leaching of heavy metals like Pb, Cd, Mg, Cr and Sb into food simulants from plastic cups at 70 °C after 2 h of contact time. Studies conducted by [13] reported the leaching of heavy metals into hot water served in plastic cups at 95 °C after 15 min. But none of the studies found out the leaching rate of heavy metals into food products within the real-time consumption (the actual time people consume a food in single-use plastic cups), let alone the potential risk assessment. People don't commonly consume the above-mentioned foodstuffs from plastic cups after one or more hours. So, studies reporting leaching rate of heavy metals from plastic or plastic cups are impractical and not representing the actual scenario. As leaching of heavy metals into food in real-time consumption, generally less than 10 min, is the only way to assess the risk associated with these metals, our study aimed at initially constructing evidence of the leaching rate of heavy metals within the time people generally consume a product such as tea, carbonated beverage and milk products served in plastic cup and assessing the potential carcinogenic and non-carcinogenic risks.

## 2 Material and methods

### 2.1 Sample collection and preparation

Twenty plastic cups of 250 mL capacity were purchased and selected at random from various shops in Jashore, Bangladesh. These recyclable plastic cups were made of polystyrene material. To the best of our knowledge, no existing health permit has been used to prepare this plastic in Bangladesh. Dry tea powder, milk, sugar, and carbonated beverages were also bought from the local market in Jashore City, Bangladesh. Using 4 g of dry tea powder, 8 g sugar, and 150 mL of hot water, tea was prepared. With the addition of 8 g sugar with 200 mL milk, Lassi (a popular milk-based local beverage) was produced according to the traditional recipe.

### 2.2 Ashing of the plastic cups, sugar and dry tea powder

The ashing of the plastic cups and other raw materials followed the technique described by [14]. Initially, the cups are fragmented into smaller segments and assessed using an electronic scale. The broken pieces of the plastic cup were thereafter placed in containers called crucibles and subjected to a temperature of 550 °C for a duration of 30 min in a muffle furnace. After the cups were burned, the crucibles were placed in a desiccator.

### 2.3 Preparation of the sample drinks

The study examined three distinct categories of sample beverages, as mentioned earlier. Each sample (tea, carbonated beverage, and lassi) was placed in plastic cups and kept for 0, 5, and 10 min. Following each time period, 10 mL of each beverage was transferred using a pipette into test tubes for subsequent digestion. Additionally, three control samples were prepared for examination and comparison, ensuring they did not come into close touch with the plastic cups.

### 2.4 Digestion of the samples

Digestion of the samples were performed as described by [15]. Ten mL of each sample (including the previously acquired liquid sample) were digested using 6 mL of concentrated nitric acid and 2 mL of concentrated hydrochloric acid and cooked on a hot plate at 90 °C with agitation for 30 min under a fume hood. The resulting transparent solutions were cooled, filtered with Whatman 41 filter paper, and then diluted with deionized water to reach the 50 mL level in a volumetric flask. The samples were then delivered to the laboratory for examination of heavy metals using Graphite Furnace Atomic Absorption Spectrophotometry (GFAAS).

### 2.5 Instrumentation of GFAAS

Each of the produced filtrates was examined using an Agilent 240Z Graphite Furnace Atomic Absorption Spectrophotometer (GFAAS). The metal concentrations were measured using an oxy-acetylene-argon gas flame, specifically at the appropriate wavelengths for each metal (283.3, 357.9, 228.8, and 324.8 nm for lead, chromium, cadmium, and copper, respectively). The operating conditions of the Agilent 240Z AAS are presented in Table 1.

**Table 1:** Agilent 240Z GFAAS instrument operating conditions.

Parameter	Setting			
Element	Pb	Cd	Cr	Cu
Instrument mode	Absorbance	Absorbance	Absorbance	Absorbance
Calibration mode	Concentration	Concentration	Concentration	Concentration
Measurement mode	Peak height	Peak height	Peak Height	Peak Height
Wavelength (nm)	283.3	228.8	357.9	324.8
Lamp type	UltrAA lamp	Hollow cathode lamp	Hollow cathode lamp	UltrAA Lamp
Lamp current (mA)	10	4	7	7
Slit width (nm)	0.5	0.5	0.2	0.2
Replicates	2	2	2	2
Calibration standards (μg/L)	10, 30, 50	1, 2, 3	2, 4, 8	10, 25, 50
Sample volume (μL)	10	10	10	10
Sample introduction	Auto-mix	Auto-mix	Auto-mix	Auto-mix
Background correction	On	On	On	On
Modifier	Premixed 2000 μg/mL NH_4_H_2_PO_4_ + 120 μg/mL Mg(NO_3_)_2_	2000 mg/L NH_4_H_2_PO_4_	300 mg/L Pd	150 mg/L Pd
Modifier injection Type	Co-injection	Co-injection	Co-injection	Co-injection
Modifier volume (μL)	5	5	5	5

The calibration standards were purchased from Sigma-Aldrich. These standards were made by diluting 1000 ppm analytical-grade stock solutions of lead, copper, cadmium, and chromium salts. Dilutions were performed using ultrapure water (18.2 MΩ cm). The standard calibration curves prepared for Pb, Cd, Cr, and Cu were ranging from 0 to 100 μg/L, 0–5 μg/L, 0–20 μg/L, and 0–1 μg/L respectively. The calibration curves were prepared considering the EU legislation [16]. All the cases yielded a correlation coefficient of 0.999. To set the detection limit, only 10 μL of sample were calculated from 7 replicate analyses of a 0.3 ppb standard solution for Pb, 0.05 ppb standard solution for Cd, 0.1 ppb standard solution for Cr, and 1 ppb standard solution of Cu. In order to verify the calibration procedure, a certified reference material of NIST 1567b Wheat Flour (Pb and Cd) and DUWF-1 (Cr and Cu) Durum Wheat Flour by AAS and the % recovery rate was presented in Table 2.

**Table 2:** Certified reference material of NIST 1567b Wheat Flour (Pb and Cd) and DUWF-1 (Cr and Cu) Durum Wheat Flour by GFAAS and their % recovery.

Element	Certified value (ppb)	Measured value (mean, n = 3) (ppb)	Standard deviation (ppb)	Recovery (%)
Pb	10.4 ± 2.4	10.9	1.4	105
Cd	25.4 ± 0.9	23.4	0.22	92
Cr	23.0 ±9.0	21.5	2.1	93
Cu	170 ± 80	172	7.5	101

### 2.6 Heavy metal analysis using GFAAS

The presence of heavy metals in all 17 samples was determined using an graphite furnace atomic absorption spectrophotometer. The equation provided was utilized to convert the instrument readings of micrograms per litre (μg/L) for each metal to parts per million (ppm) or milligrammes per kilogramme (mg/kg). This conversion allowed for the determination of the average standard deviation (SD) concentrations of the metals that leached into the drinks.

Metalconcentration(inppm)=(Vol×α×df)/(1000×wt)

where Vol is the sample volume utilized, df is the dilution factor, and wt is the sample weight, α is the metal instrument reading in μg/L.

### 2.7 Health risk assessment

#### 2.7.1 Non-carcinogenic risk

The non carcinogenic risk of consuming the drinks in plastic cup at different time intervals were evaluated using the following formula,

$$\text{Target}\;\text{Hazard}\;\text{Quotient}\;\left(\text{THQ}\right)=\left({\text{EF}}_{r}\times \text{ED}\times \text{FIR}\times C\right)/\left(\text{RfD}\times \text{BW}\times \;\text{ATn}\right)\times 10^{-3}$$

Here, EF_r_ = Exposure Frequency (365 days/year); ED = Exposure Duration (60 years); FIR is the food ingestion rate (g/day); C is the heavy metal concentration in tea, carbonated drinks, and lassi (mg/kg); BW is the average adult body weight (60 kg); ATn is the average exposure time for non-carcinogens (EF × ED) (365 days/year for 60 years (i.e., ATn = 21,900 days)); RfD is the reference dose for metals where, Cr = 0.003, Cu = 0.03, Cd = 0.001 and Pb = 0.2 [17].

#### 2.7.2 Hazard index

The total of all hazard quotients (THQ) determined for individual heavy metals for a specific exposure pathway is termed as the chronic hazard index (HI), which is used to assess the potential danger to human health from exposure to multiple heavy metals and is determined by using the following formula,

HI=THQ(Pb)+THQ(Cr)+THQ(Cu)+THQ(Cd)

#### 2.7.3 Carcinogenic risk

The Incremental Lifetime Cancer Risk method was used to calculate the potential cancer risks in examined raw ingredients and in the prepared tea, carbonated drinks, and lassi due to the consumption of carcinogenic heavy metals and calculated by the formula used by [18].

ILCR(IncrementalLifetimeCancerRisk)=CDI×CSF

Where CDI represents the chronic daily intake of a chemical carcinogen and expressed as mg/kg BW/day that indicates the average daily dose of exposure to the chemical carcinogen over the course of a lifetime. The cancer slope factor (CSF), a contaminant-specific risk created by a lifetime average dosage of 1 mg/kg BW/day, is used to calculate the ILCR [19]. The range between 10^−6^ and 10^−4^ is regarded as the level of acceptable cancer risk (ILCR) for regulatory purposes [20]. The CSF for the Pb, Cr, Cd, and Cu are 8.5, 41, 6.1 and 0 mg/kg BW/day respectively [21]. The following equation was used to compute the CDI value, and the literature's recommended CSF values for carcinogenic heavy metals were employed [22].

$$\text{CDI}=\left(\text{EDI}\;\times {\text{EF}}_{r}\times {\text{ED}}_{\text{tot}}\right)\;/\text{AT}$$

where EDI indicates the estimated daily intake, which includes lassi, tea, and carbonated beverages as sources of metal; while AT is the period of exposure for carcinogenic effects (60 years life time). EF_r_ is the exposure frequency (365 days/year); ED_tot_ is the exposure duration (60 years), typical lifetime for Bangladeshis [19]. The following equation was used to compute the total cancer risk due to intake of a particular variety of tea, carbonated beverage, or lassi after exposure to several carcinogenic heavy metals, which was assumed to be equal to the sum of the individual hazards from each heavy metal [18].

$$\text{Total}\;\text{ILCR}={\text{ILCR}}_{1}+{\text{ILCR}}_{2}+\cdots +{\text{ILCR}}_{n}$$

where the number of cancer-causing heavy metals (n) is 1, 2, …, n.

### 2.8 Statistical analysis

All the analysis were done in triplicates. The statistical analysis was conducted using IBM SPPS software version 25. A one-way analysis of variance (ANOVA) was performed to compare the means of different cases, with a significance level set at p < 0.05. A Pearson's correlation coefficient analysis was conducted to determine the correlation between variables (one-tailed) at a significance level of p < 0.01, in order to investigate the sources of heavy metal migration. The data's normality was evaluated using the Shapiro-Wilk test.

## 3 Results and Discussion

### 3.1 Total concentration of heavy metals (Pb, Cr, Cd, Cu) in plastic cups and food samples

It is evident from Table 3 that traces of heavy metals (Pb, Cr, Cd, Cu) were found to be present in plastic cups and in other raw ingredients used in this study. The data collected exhibited a normal distribution, as evidenced by a p-value of 0.211 in the Shapiro-Wilk test. This suggests that the data followed a normal distribution with a significance level of 0.05. Heavy metals in water were detected for both before and after boiling stage and the concentration of heavy metals after boiling increased, but this increases were not of significant difference at p < 0.05. These increases in heavy metal concentration were due to the loss of water molecules as vapor during boiling.

**Table 3:** Concentration of heavy metals in plastic cup, milk, water, dry tea and sugar.

Sample	Concentration of heavy metal (mg/kg) (n = 3)			
	Pb	Cr	Cu	Cd
Plastic Cup	0.2351 ± 0.03^a^	0.1342 ± 0.01^a^	0.2068 ± 0.03^a^	0.0031 ± 0.006^a^
Water (before boiling)	0.0231 ± 0.04^b^	0.0526 ± 0.03^b^	0.0367 ± 0.03^c^	0.0024 ± 0.002^b^
Water (after boiling)	0.0235 ± 0.02^b^	0.0529 ± 0.02^b^	0.0371 ± 0.04^c^	0.0026 ± 0.003^b^
Milk	0.0222 ± 0.01^b^	0.0191 ± 0.02^c^	0.0323 ± 0.04^c^	0.0012 ± 0.003^c^
Sugar	0.1233 ± 0.04^c^	0.0312 ± 0.04^d^	0.0345 ± 0.05^c^	0.0009 ± 0.006^c^
Tea Powder	0.5797 ± 0.04^d^	0.617 ± 0.04^b^	0.5416 ± 0.03^b^	0.0002 ± 0.002^d^

*Values are presented as mean ± SD. n represents the number of replicates. Values with different alphabets within a column indicates significant difference at p < 0.05.

**Fig. 1:**
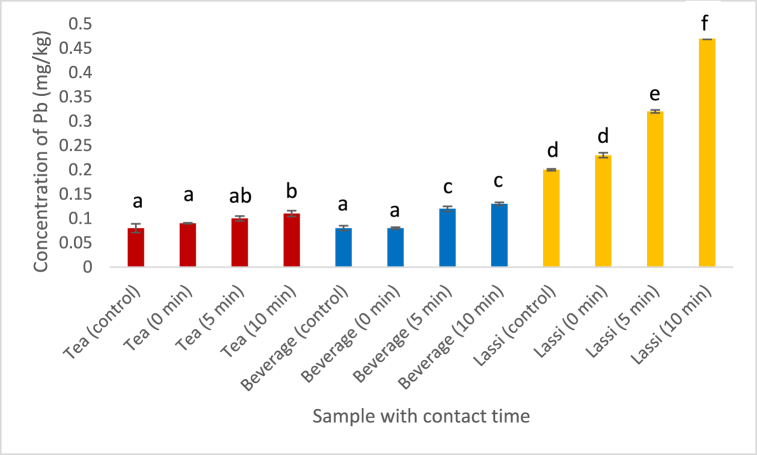
Effect of contact time on the leaching of Pb into the foodstuffs. (Values with different alphabet indicates significant difference at p < 0.05. Analysis was done in triplicates.)

Lead (Pb) is a significant carcinogenic substance when found in food and consumed on a daily or yearly basis. It was determined that the studied samples contained a far too high percentage of Pb compared to [23]. The plastic cup contained the highest concentration of lead (0.2351 mg/kg) of all the raw ingredient samples in Table 3. Tea powder also contains a substantial quantity of lead (0.2229 mg/kg). Both the Pb concentration was over the maximum allowable threshold of 0.02 mg/kg [23]. In three selected and prepared liquid drinks, a rising trend of lead content was detected as contact time increased (Fig. 1). This trend is particularly significant for lassi (0.2059–0.4720 mg/kg) samples. Similar findings were observed in the study of [24] and they found that Pb concentration reached 0.0054 mg/kg from 0.0011 mg/kg after 15 min of contact time into water. They also found the migrated heavy metal concentration were higher than the permissible limit stated by [25] when the food samples are placed in the plastic packaging for longer period.

**Fig. 2:**
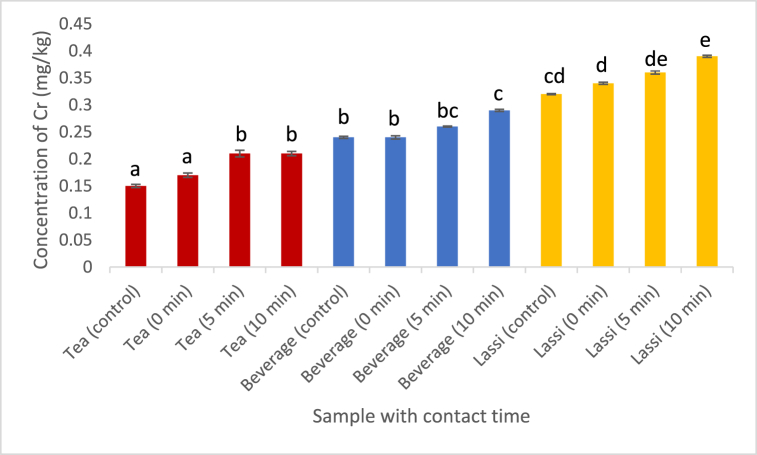
Effect of contact time on the leaching of Cr into the foodstuffs. (Values with different alphabet indicates significant difference at p < 0.05. Analysis was done in triplicates.)

Cr (Chromium) was detected in all of the samples analyzed in our investigation. The examined plastic cups contained a concentration of Cr greater than 0.1 mg/kg, as stated by [26]. The tea powder contained a comparable proportion of Cr. The concentration rose as time progressed. The Cr concentration that leached from the hot tea and cold lassi samples rose approximately identically with increasing contact duration. But the carbonated beverage samples showed elevated concentration of Cr with respect to time of contact when compared with the tea and lassi samples (Fig. 2). This could be due to the acidic composition of carbonated beverages. [27] reported the same kind. The tea powder had a similar amount of chromium. The concentration increased over time. The concentration of Cr that leached from both the hot tea and cold lassi samples increased in a similar manner as the contact period increased. However, the carbonated beverage samples exhibited a higher concentration of Cr with time compared to the tea and lassi samples. It was noted that acidic liquids expedite the process of chromium leaching from old plastic cups in comparison to neutral or alkaline beverages.

**Fig. 3:**
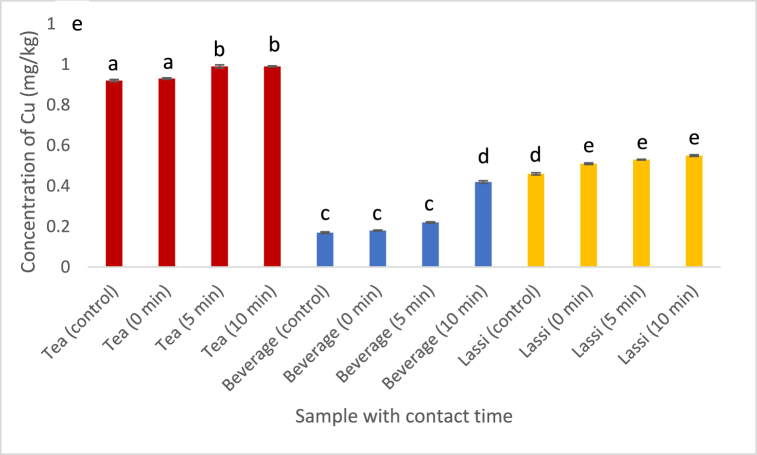
Effect of contact time on the leaching of Cu into the foodstuffs. (Values with different alphabet indicates significant difference at p < 0.05. Analysis was done in triplicates.)

[28] reported the permissible concentration of copper in food should not go beyond 1 mg/kg. We noticed an increase in the concentration of Cu in tea (from 0.92 to 0.99 mg/kg) as the contact time with plastic cups increased (Fig. 3). In our study, we found that Cu is far more concentrated in plastic cups than in water, milk, or sugar. Copper ions (Cu^2^⁺) exhibit significant mobility in aqueous solutions. This implies that they have the ability to readily separate from the plastic substance and disintegrate into the food or liquid it comes into touch with. Hence, the probability of Cu leaching and migrating from the plastic cups is higher compared to that from the basic materials.

**Fig. 4:**
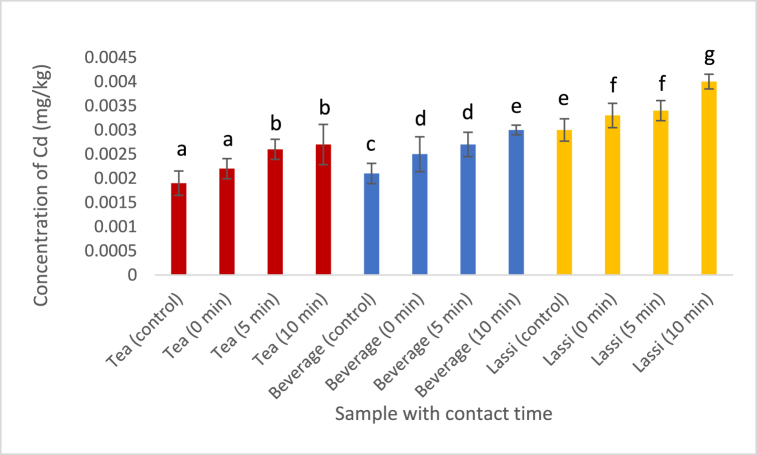
Effect of contact time on the leaching of Cd into the foodstuffs. (Values with different alphabet indicates significant difference at p < 0.05. Analysis was done in triplicates.)

Cadmium (Cd), a highly significant food-borne toxin, was detected in all samples except for water, milk, and sugar. Fig. 4 represents the concentration of the heavy metals detected in the samples at different degrees of contact time. Hot tea and carbonated beverage contained Cd in higher concentration than that of the standard mentioned in [29]. But lassi had the highest concentration of Cd with the increase in contact time with the plastic cups (>0.0025 mg/kg as per EFSA standard). Since no traces of Cd were found in the milk, water, and sugar used and analyzed in this study, it can be concluded that the concentration of Cd that was detected in the plastic cups originated from leaching and migration.

Plastic cups, because of the way they are made, frequently contribute to the release of heavy metals. The use of pigments and adhesives in their manufacturing process increases the probability that they may serve as a potential origin of heavy metal pollution [30]. Added to that, temperature plays a significant role in the heavy metal leaching from plastic cups. [31] found the same causes of heavy metals leaching along with the effect of different simulants.

A comparison of the heavy metals found in the samples in contact with plastic cup used in this study and by different study worldwide was depicted in Table 4. Each sample seemed to be polluted with heavy metals at variable amount. Thus, it strengthened the findings of this study.

**Table 4:** Comparison of the concentration of heavy metals (Pb, Cd, Cr and Cu) found worldwide and in this study.

Sample in contact with plastic	Name of heavy metal migrated	Migration of heavy metals with respect to contact time in min (mg/kg)				Reference
		<1	5	10	>10	
Tea	Pb	0.092	0.1107	0.1129	–	This study
	Cd	0.0024	0.0030	0.0030	–	
	Cr	0.1748	0.0270	0.2157	–	
	Cu	0.9422	1.0233	0.9843	–	
Beverage	Pb	0.0869	0.1284	0.1385	–	
	Cd	0.0028	0.0028	0.0034	–	
	Cr	0.3757	0.3372	0.4275	–	
	Cu	0.1703	0.2377	0.4286	–	
Lassi	Pb	0.2363	0.3219	0.4720	–	
	Cd	0.0033	0.0034	0.0040	–	
	Cr	0.2271	0.3024	0.2797	–	
	Cu	0.5085	0.5391	0.5347	–	
–	Pb	–	–	–	2.00	[16]
	Cr	–	–	–	1.00	
–	Pb	–	–	–	2.00	[32]
	Cd	–	–	–	2.00	
Milk	Pb	–	–	–	0.02 ± 0.004 (30 min), 0.337 ± 0.008 (60 min)	[30]
	Cd	–	–	–	–	
	Cr	–	–	–	0.11 ± 0.004 (30 min), 0.147 ± 0.004 (60 min)	
	Cu	–	–	–	0.11 ± 0.002 (30 min), 0.24 ± 0.001 (60 min)	
Dry tea	Pb	–	–	–	0.053 ± 0.001 (30 min), 0.093 ± 0.001 (60 min)	[30]
	Cd	–	–	–	–	
	Cr	–	–	–	0.193 ± 0.002 (30 min), 0.354 ± 0.005 (60 min)	
	Cu	–	–	–	0.47 ± 0.003 (30 min), 3.61 ± 0.022 (60 min)	
White cheese	Pb	–	–	–	7.8	[33]
	Cd	–	–	–	0.4	
	Cr	–	–	–	–	
	Cu	–	–	–	4.6	
	Cu	–	–	–	0.45	
Hot water	Pb	–	–	–	15.5 (15 min)	[34]
	Cd	–	–	–	3.00 (15 min)	
	Cr	–	–	–	0.00285 (15 min)	
	Cu	–	–	–	0.00233	

### 3.2 Health risk assessment

[35] stated that risk assessment for bio-accumulated heavy metals was estimated using target hazard quotient (THQ), hazard index (HI) and target ILCR (Incremental lifetime cancer risk). These factors consider the frequency and length of exposure, the average weight of the body, and the oral reference dosage, in addition to the amount of contaminant ingested (RfD).

#### 3.2.1 Non-carcinogenic risk assessment

[15] stated that the magnitude of the effect was proportional to the sum of the multiple metal exposures and that similar working mechanism linearly affects the target organ. The calculated HI was compared to standard levels: the population was assumed to be safe when HI < 1 and in a level of concern when 1 < HI < 5.

From Table 5, it could be seen that, none of the products exhibited a Target Hazard Quotient (THQ) with a risk level greater than 1. All of the Total Hazard Quotient values are below 1, indicating that the calculated Health Index values were less than 1. Based on the Health Index results, it can be concluded that none of the samples with the previously mentioned concentration of heavy metals posed a non-carcinogenic risk. This could be attributed to the insufficient duration of contact between the liquid sample and the plastic, which resulted in the absence of any non-carcinogenic hazards. Similar finding were observed by [36] when they studied the effect of the contact time with Polyethylene or Polystyrene food containers.

**Table 5:** THQ and HI for population through the consumption of different types of Carbonated beverages as tea, lassi, carbonated beverage by using plastic cups.

Sample	THQ For				HI
Name	Pb	Cr	Cd	Cu	
Tea control	9.95 × 10^−6^	1.30 × 10^−3^	4.86 × 10^−5^	7.84 × 10^−4^	0.0021
Tea< 1 min	1.15 × 10^−5^	1.47 × 10^−3^	5.62 × 10^−5^	7.93 × 10^−4^	0.0023
Tea 5 min	1.34 × 10^−5^	1.81 × 10^−3^	6.65 × 10^−5^	8.48 × 10^−4^	0.0027
Tea 10 min	1.52 × 10^−5^	1.85 × 10^−3^	6.90 × 10^−5^	8.51 × 10^−4^	0.0027
Carbonated beverage control	1.18 × 10^−4^	2.25 × 10^−2^	5.82 × 10^−4^	1.64 × 10^−3^	0.0248
Carbonated beverage< 1 min	1.21 × 10^−4^	2.26 × 10^−2^	6.93 × 10^−4^	1.74 × 10^−3^	0.0251
Carbonated beverage 5 min	1.78 × 10^−4^	2.43 × 10^−2^	7.49 × 10^−4^	2.08 × 10^−3^	0.0273
Carbonated beverage 10 min	1.93 × 10^−4^	2.74 × 10^−2^	8.32 × 10^−4^	3.89 × 10^−3^	0.0323
Lassi control	8.35 × 10^−4^	8.80 × 10^−2^	2.42 × 10^−3^	1.25 × 10^−2^	0.1039
Lassi< 1 min	9.56 × 10^−4^	9.17 × 10^−3^	ND	1.38 × 10^−2^	0.0240
Lassi 5 min	1.29 × 10^−3^	9.69 × 10^−2^	ND	1.43 × 10^−2^	0.1120
Lassi 10 min	1.90 × 10^−3^	1.06 × 10^−1^	ND	1.48 × 10^−2^	0.1237
Water	3.85 × 10^−3^	5.84 × 10^−1^	ND	4.70 × 10^−3^	0.6290
Milk	Nd	2.89 × 10^−3^	ND	4.55 × 10^−4^	0.0030
Tea powder	7.41 × 10^−5^	5.26 × 10^−3^	5.11 × 10^−6^	4.61 × 10^−4^	0.0058
Sugar	ND	2.05 × 10^−2^	ND	1.54 × 10^−3^	0.0256

*ND **=** Not detected.

#### 3.2.2 Carcinogenic risk assessment

The computed incremental lifetime cancer risk (∑ILCR) for Cr, Cu, Cd, and Pb through the studied food products are presented in Table 6 above. [17] recommended the safe limit for cancer risk is below about 1 chance in 1,000,000 lifetime exposure (ILCR <10^−6^) and threshold risk limit (ILCR >10^−4^) for chance of cancer is above 1 in 10,000 exposure where remedial measures are considerable and moderate risk level (ILCR >10^−3^) is above 1 in 1000 where public health safety consideration is more important.

**Table 6:** ILCR and cumulative cancer risks (∑ILCR) for the adult through consumption of different type of Carbonated beverages.

Sample Name	CDI for				CSF				ILCR
	Cr	Cu	Cd	Pb	Cr	Cu	Cd	Pb	
Plastic (Control)	–	–	–	–	–	–	–	–	–
Tea (control)	0.0039	0.0235	4.86 × 10^−5^	0.0199	41	0	6.1	8.5	2 × 10^−1^
Tea (<1 min)	0.0044	0.0238	5.62 × 10^−5^	0.0023	41	0	6.1	8.5	2.1 × 10^−1^
Tea (5 min)	0.0054	0.0254	6.65 × 10^−5^	0.0026	41	0	6.1	8.5	2.2 × 10^−1^
Tea (10 min)	0.0055	0.0255	6.90 × 10^−5^	0.0030	41	0	6.1	8.5	2.2 × 10^−1^
Carbonated beverage (control)	0.0675	0.0494	5.82 × 10^−4^	0.0237	41	0	6.1	8.5	3 × 10^0^
Carbonated beverage (<1 min)	0.0679	0.0523	6.93 × 10^−4^	0.0243	41	0	6.1	8.5	3 × 10^0^
Carbonated beverage (5 min)	0.0729	0.0624	7.49 × 10^−4^	0.0358	41	0	6.1	8.5	3 × 10^0^
Carbonated beverage (10 min)	0.0822	0.1167	8.32 × 10^−4^	0.0386	41	0	6.1	8.5	4 × 10^0^
Lassi (control)	0.2642	0.3771	2.42 × 10^−3^	0.1671	41	0	6.1	8.5	1 × 10^1^
Lassi (<1 min)	0.2753	0.4159	0	0.1913	41	0	6.1	8.5	1 × 10^1^
Lassi (5 min)	0.2909	0.4297	0	0.2599	41	0	6.1	8.5	1 × 10^1^
Lassi (10 min)	0.3209	0.4461	0	0.3809	41	0	6.1	8.5	2 × 10^1^
Water	1.7533	1.2233	0	0.77	41	0	6.1	8.5	8 × 10^1^
Milk	0.0086	0.0136	0	0	41	0	6.1	8.5	4 × 10^−1^
Tea Powder	0.0157	0.0138	5.11 × 10^−5^	0.0148	41	0	6.1	8.5	8 × 10^−1^
Sugar	0.0132	0.0242	0	0	41	0	6.1	8.5	3 × 10^−8^

Among the samples, Lassi had the highest level of estimated risk. According to Table 6, the ILCR values of lassi increased from 0 to 10 min. Moreover, the water utilized possessed a significant potential for harm. The carbonated beverage also exhibited a similar pattern of increasing ILCR values, rising from 3 × 10^0^ to 4 × 10^0^ within 10 min. The ILCR value of tea did not exhibit any substantial alterations with variations in contact time.

Immediate action is required to mitigate the leaching of heavy metals caused by the excessive utilization of disposable plastic cups. Therefore, it is crucial to regulate the level of food exposure in order to protect the population from the risk of cancer.

### 3.3 Pearson's correlation coefficient (r) analysis of sources of different heavy metal migration

Pearson's correlation coefficient analysis (Table 7) revealed that all the samples have one source of heavy metal migration and that was plastic cups in which they stayed for different time intervals. The correlation analysis revealed that the r values of all the samples, excluding the controls and tea powder, exhibited a significant correlation (p < 0.01) with plastic cups. Furthermore, the tea samples that had no contact with the plastic cup for 0 min demonstrated a significant correlation with all the other tea samples. These findings suggest that all the tea samples contained a common source of heavy metal, namely the plastic cups. Conversely, the other raw ingredients displayed either insignificant or negative correlations with the samples.

**Table 7:** Correlation coefficient analysis of different sources of heavy metal migration.

	Plastic	Water	Milk	Sugar	TP	TC	T0	T5	T10	BC	B0	B5	B10	LC	L0	L5	L10
Plastic	1*	−0.113	−0.068	−0.595	−0.876	−0.479	0.980*	0.987*	0.999*	−0.484	0.798*	0.821*	0.823*	−0.716	0.743*	0.844*	0.953*
Water	−0.113	1*	−0.898	−0.457	−0.348	−0.155	−0.174	−0.197	−0.192	−0.813	−0.800	−0.703	−0.541	−0.466	−0.417	−0.319	−0.157
Milk	−0.068	−0.898	1*	−0.084	−0.417	−0.217	−0.196	−0.171	−0.172	−0.732	−0.706	−0.620	−0.298	0.258	0.209	0.170	0.114
Sugar	−0.595	−0.457	−0.084	1*	−0.462	−0.393	−0.397	−0.394	−0.382	−0.082	−0.094	−0.009	−0.206	−0.088	−0.062	−0.103	−0.364
TP	−0.876	−0.348	−0.417	−0.462	1*	0.373	0.384	0.402	0.412	−0.811	−0.814	−0.883	−0.744	−0.805	−0.806	−0.872	−0.933
TC	−0.479	0.155	−0.217	−0.393	0.373	1*	0.299	0.398	0.398	−0.451	−0.485	−0.530	−0.838	−0.823	−0.836	−0.778	−0.632
T0	0.680	0.174	−0.196	−0.397	0.384	0.299	1*	0.999*	0.999*	0.469	0.503	0.546	0.848	0.833	0.844	0.786	0.638
T5	0.787	0.197	−0.171	−0.394	0.402	0.398	0.999*	1*	0.999*	0.492	0.526	0.568	0.862	0.846	0.857	0.798	0.649
T10	0.499	0.192	−0.172	−0.382	0.412	0.298	0.999*	0.999*	1*	0.494	0.528	0.572	0.864	0.850	0.861	0.804	0.658
BC	−0.484	−0.813	−0.732	−0.082	−0.811	−0.451	−0.469	−0.492	−0.494	1*	0.299	0.285	0.260	−0.846	−0.819	−0.788	−0.701
B0	0.898*	−0.300	−0.706	−0.094	−0.814	−0.485	−0.503	−0.526	−0.528	0.399	1*	0.988*	0.879*	−0.865	−0.840	−0.807	−0.717
B5	0.821*	0.703	−0.620	−0.009	−0.883	−0.530	−0.546	−0.568	−0.572	−0.985	0.988*	1*	0.906*	−0.909	−0.890	−0.877	−0.812
B10	0.823*	0.300	−0.298	−0.206	−0.744	−0.838	−0.848	−0.862	−0.864	−0.860	0.879*	0.906*	1*	−0.992	−0.985	−0.945	−0.827
LC	−0.716	0.466	0.258	−0.088	−0.805	−0.823	−0.833	−0.846	−0.850	−0.846	−0.865	−0.906	−0.992	1*	0.398	0.478	0.405
L0	0.943*	0.417	0.209	−0.062	−0.806	−0.836	−0.844	−0.857	−0.861	−0.819	−0.840	−0.890	−0.985	0.398	1*	0.985*	0.905*
L5	0.844*	0.319	0.170	−0.103	−0.872	−0.778	−0.786	−0.798	−0.804	−0.788	−0.807	−0.877	−0.945	0.978*	0.985*	1	0.963*
L10	0.843*	0.157	0.114	−0.364	−0.933	−0.632	−0.638	−0.649	−0.658	−0.701	−0.717	−0.812	−0.827	0.905*	0.905*	0.963*	1

*Significant correlation exists at p < 0.01.

### 3.4 Relation of contact time and other factors on the leaching behavior of the heavy metals from plastic cups

Our study attempted to establish the relationship between the leaching behavior of heavy metals with the contact time of meals (particularly liquid foods) with the plastic cups. Other factors such as temperature, pH etc. of the food and their effects during real-time consumption was also studied.

Pb is more susceptible to leaching at lower pH levels. The study of [32] reported the same. They found that at low pH (<7.00), the concentration of Pb that leached from plastic was on average 80 ppb. As carbonated beverage is more acidic compared to the other samples, this could be a cause for highest leaching amount of Pb. Acidic conditions hasten the deterioration of the plastic surface. As the surface degrades, it becomes increasingly porous or develops microfractures, which create channels for lead ions to leach out more easily. Acidic surroundings facilitate chemical reactions. Acids promote the disintegration of chemical bonds in the plastic substance, enabling lead ions to separate and move into the surrounding solution. Lead, a metallic element, undergoes chemical reactions with different compounds when specific circumstances are fulfilled. Under acidic conditions, such as in low pH situations, the lead ions undergo a reaction with the constituents of the plastic, resulting in their release into the surrounding environment [17]. Our investigation found that plastic released the largest concentrations of Pb (lead) and Cr (chromium) in carbonated beverages, with tea and lassi following after. [32] reported the same leaching behavior of Cr in low pH. As chromium is resistant to corrosion and discoloration and is consequently employed in high concentrations in the production of plastics, according to [37], the high concentration of Cr in the cups may be the result of the excessive use of chromium compounds.

Cu is used in plastic cups during molding as it is highly heat resistant. [30] found the relationship of temperature on the leaching of Cu from plastic cups. They reported that, higher temperatures can accelerate the leaching of copper from plastic containers. [38] also found the temperature effect on the leaching quantity of Cu. The high Cu level in the tea samples was due to the tea's high temperature relative to the other samples we studied. As the temperature increases, the molecules in both the plastic substance and the food or beverage get more energy. The elevated kinetic energy can enable the transfer of copper ions from the plastic material into the meal or drink [39]. Elevated temperatures typically enhance the solubility of compounds. Consequently, the likelihood of copper ions being released from the plastic and dissolving into the food or beverage increases at higher temperatures, resulting in a larger degree of leaching. In addition, the investigation revealed that the concentration of Cu in tea reduced as time passed, and a decreasing order of 0.9923 mg/kg to 0.9902 mg/kg was detected between 5- and 10 min tea samples as the samples cooled. This establishes the effect of temperature on Cu leaching behavior from plastic cups into meals.

### 3.5 Health risk assessment of heavy metals leached from plastic cups into foodstuffs

The concentration of heavy metals in food products often varies depending on the accumulation capabilities and contact times of each item [40].

[30] reported in their study that, the plastic cups leached significant (P < 0.05) concentrations of chromium, copper, cadmium and lead into food products at contact times of 10 than 0 or 5 min. This occurs because diffusion promotes the extraction of metals from the polymer matrix. The accumulation of lead, chromium, and copper in drinks from plastic is influenced by the diffusion flux, which is determined by the duration that food components remain in the cup. Similar finding were reported by [41]. [42] also reported similar findings when they detected the effect of temperature and contact time on antimony leaching out of PET containers.

Our analysis found no non-carcinogenic risk due to the leaching of heavy metals from plastic cups. Contrarily, other samples exhibited moderate to severe carcinogenic risks. The likelihood of carcinogenic danger is as follows: lassi > carbonated beverage > tea from the control to the contact time of 10 min. Milk and tea powder also exhibited carcinogenic properties. Milk and milk products are very vulnerable to heavy metal toxicity as a result of the consumption of contaminated feed and water by animals, as well as the use of fertilizers made from polluted crops and the collection of feed from industrial areas with high levels of pollution [43]. This study revealed the presence of the heavy metals Pb, Cr, and Cu in milk. Although there is no presence of Cd in the milk, it still poses a significant carcinogenic risk. Undoubtedly, the presence of the other two metals in the milk sample can be attributed to their migration from the plastic cup. This migration resulted in an increase in the toxicity of heavy metals, which function as carcinogens, in the lassi samples after 10 min of contact time. Tea is a heated beverage used in this case, and as the temperature increases, the extraction of toxic metals from plastic intensifies. Temperature speeds up the aging process, which in turn speeds up the leaching of heavy metals [44]. At first, it induces the movement of any existing small-sized additional substances in old materials. Previous studies conducted by researchers [41,45] have shown similar findings. [39] reported that, heavy metal leaching was affected in great extent when the pH of the product tended to be acidic (4–7) than to a pH of 8–10. This might be the case for carbonated beverage when it stayed for a longer period of time (for up to 10 min). When carbonated beverages are consumed for a prolonged period of time and have a low pH (2.6–2.7), the Incremental Lifetime Cancer Risk (ILCR) increases, making them possibly carcinogenic.

## 4 Conclusion

Following the outbreak of the COVID-19 pandemic, there has been a surge in the use of disposable plastic cups owing to their hygienic properties and ease of transportation. Nevertheless, the process of chemicals and heavy metals seeping out of plastic has rendered it perilous to the well-being of consumers. The aim of our study was to investigate the process of heavy metals being released from plastic cups into different food products over the course of actual eating. The study utilized graphite furnace atomic absorption spectrophotometry (GFAAS) to discover that all samples contained varying quantities of heavy metals following a specific duration of exposure to plastic cups. Heavy metals leaching from the samples include the following manner: Cu > Pb > Cr > Cd. Lassi has the most elevated levels of leached Pb and Cd. Cu was for tea, whereas Cr was for carbonated beverages. No samples showed any evidence of non-carcinogenic risk. Nevertheless, the carbonated beverages, lassi, and tea samples that had been in contact with plastic cups for different durations exhibited carcinogenic risk levels that were above the guideline set by the USEPA. The leaching of heavy metals was influenced by the temperature and pH of the food product to varying extents. Plastic cups are widely used and highly convenient serving utensils due to their widespread availability and user-friendly nature. Nevertheless, legislation should be implemented to forbid its widespread utilization as a result of the presence of heavy metals and their hazardous properties. Though this study investigated the effect of contact time on the heavy metals leaching behavior from plastic cups into different foodstuff, it limits only on one type of plastic (Polystyrene). Improvement can be brought by investigating the leaching behavior of heavy metals in other types of plastics. Further investigation can be carried out by replacing food constituents and adjusting the temperature, pH, and density of the meal served in plastic cups. Furthermore, further examination can be carried out to assess the release of heavy metals from plastic cups by comparing them with cups made of glass, paper, and other materials.

## Data availability statement

Data will be made available on request.

## CRediT authorship contribution statement

**B.M. Khaled:** Writing – original draft, Visualization, Validation, Supervision, Methodology, Investigation, Conceptualization. **Adda Ann Sina:** Writing – review & editing, Validation. **Md Suman Rana:** Writing – review & editing, Resources, Data curation. **S.M. Shamiul Alam:** Writing – review & editing, Visualization, Validation. **Abdullah Al Numan:** Resources, Methodology, Formal analysis. **Maria Tabassum Shammi:** Methodology, Formal analysis. **Fatima Parvin:** Methodology, Formal analysis. **Tamanna Naznin:** Writing – review & editing, Visualization. **Md Mozaffor Hossain:** Writing – review & editing, Visualization. **Refat Pervin Annana:** Writing – review & editing, Visualization.

## Declaration of competing interest

The authors declare that they have no known competing financial interests or personal relationships that could have appeared to influence the work reported in this paper.
